# Takashi Yura—in memory of an inspiring pioneer of the bacterial heat shock response

**DOI:** 10.1007/s12192-023-01355-8

**Published:** 2023-06-22

**Authors:** Bernd Bukau

**Affiliations:** Center for Molecular Biology of Heidelberg University (ZMBH), 69120 Heidelberg, Germany

Takashi Yura has been an inspiration for me right from the beginning of my career. In the 1980s, I worked as a postdoc in Graham Walker’s lab at MIT to elucidate the function and regulation of a formerly mysterious *E. coli* protein termed DnaK. Takashi’s publications on the bacterial heat shock response guided me in many ways. He started the field by the pioneering discovery of *rpoH*, the gene that encodes the heat shock promoter-specific sigma 32 subunit of RNA polymerase. He showed how *rpoH* itself is regulated and how it controls the expression of *dnaK* and other heat shock genes. He also found that the massive protein aggregation phenotype of *∆rpoH* mutants can be suppressed by the overexpression of GroEL and DnaK. His seminal findings strongly influenced my research, as they demonstrated, through elegant genetic experiments, that these chaperones and sigma 32 play a central role in protein folding. Many labs including mine have since tried to figure out how chaperones act in the folding of proteins in bacteria. Even though my research interests broadened, I kept interest in sigma 32 and the activity of DnaK in the regulation of the heat shock response. In fact, my lab recently made a discovery which fully supports Takashi’s genetic findings, and I was very much looking forward to meeting him in Japan to tell him about our findings; he would have been very pleased. Unfortunately, the corona pandemic let to cancelation of a planned visit, and now it is too late.

I encountered Takashi at conferences but also during mutual visits in Heidelberg and Kyoto. He is a kind and modest man who generously shared his knowledge and advice. His kindness was immense and went well beyond scientific issues. He showed me temples and shrines in Kyoto, and not surprisingly in light of Takashi’s personality, he brought me to the oldest Zen Buddhist temple, the Ryoan-ji with its most peaceful and thought-stimulating rock garden. The garden appears in modesty, and this exactly is so impressive that I went back several times since then. When Takashi invited me to his private house in Kyoto, I also noticed the modest yet tasteful style that he and his wife were living. At a dinner in a traditional Japanese ryokan in Kyoto, he noticed the difficulties I had in eating some of the dishes. He helped me out by saying that “even for me, this is a little bit strange food,” which relaxed the situation for me immediately. In turn, I was happy to be able to show him historic hidden places in Heidelberg, which he and his wife enjoyed during a trip in the 1990s. I will never forget his visit in Heidelberg, where we discussed the regulation of the *rpoH* gene in great detail and had a wonderful evening with him and his wife.

Takashi was respectful to everybody, especially also to young scientists, and never put himself above others. He showed immense interest for research in the purest sense. In fact, after all his successes and high-ranked positions in academia, he went back to the bench and started pipetting at an age well in his 70 s. He continued to investigate his main research theme—the regulation of the heat shock response in bacteria. I admired this a lot; not too many scientists express their genuine interest in science by going back to tiring lab work, well after retirement. I had wished that Takashi could visit us again in Heidelberg, for interactions with our students. There is no better role model for a scientist I can think of.

I am so grateful for the inspiring and pioneering scientific work you shared and the unforgettable memories of our personal encounters. Thank you, Takashi.

**Figure:**
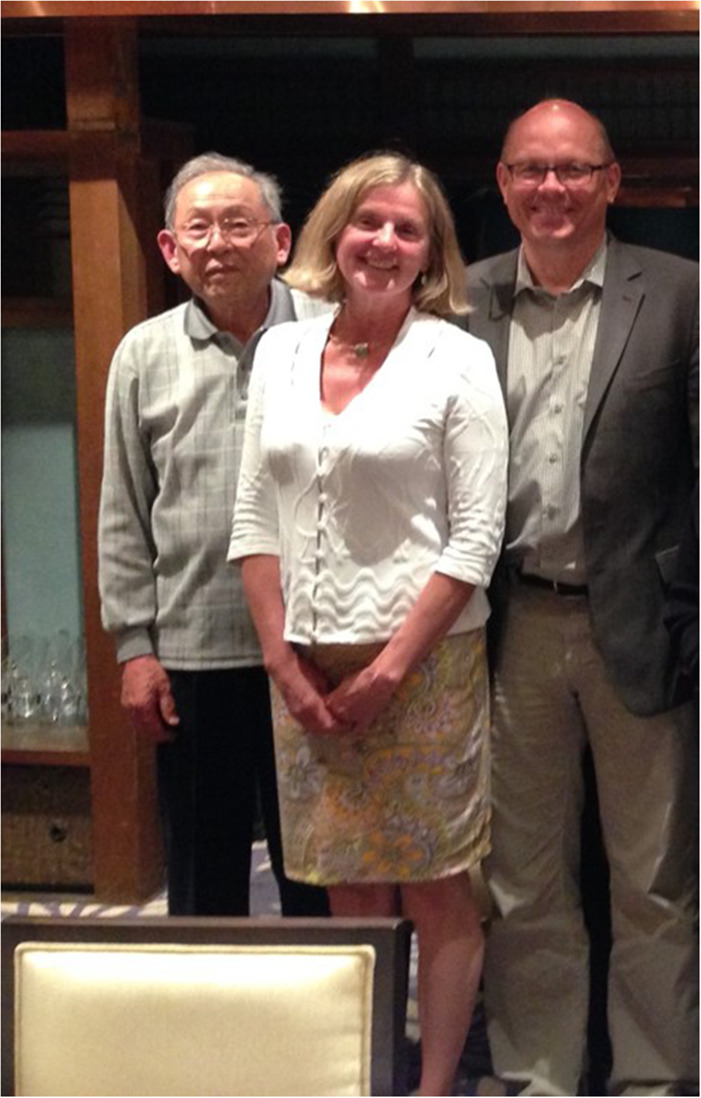
In Kyoto, Japan, 2014. Takashi Yura, Anette Bukau, and Bernd Bukau

